# Shear wave elastography predicts hepatocellular carcinoma risk in hepatitis C patients after sustained virological response

**DOI:** 10.1371/journal.pone.0195173

**Published:** 2018-04-19

**Authors:** Koichi Hamada, Satoshi Saitoh, Noriyuki Nishino, Daizo Fukushima, Yoshinori Horikawa, Shinya Nishida, Michitaka Honda

**Affiliations:** 1 Department of Minimally Invasive Surgical and Medical Oncology, Fukushima Medical University, Fukushima, Japan; 2 Department of Gastroenterology, Southern-Tohoku General Hospital, Koriyama, Japan; 3 Department of Hepatology, Toranomon Hospital, Tokyo, Japan; Universita degli Studi di Pisa, ITALY

## Abstract

**Aim:**

To evaluate the relationship between fibrosis and HCC after sustained virological response (SVR) to treatment for chronic hepatitis C (HCV).

**Methods:**

This single-center study retrospectively evaluated 196 patients who achieved SVR after HCV infection. The associations of risk factors with HCC development after HCV eradication were evaluated using univariate and multivariate Cox proportional hazards regression models.

**Results:**

Among the 196 patients, 8 patients (4.1%) developed HCC after SVR during a median follow-up of 26 months. Multivariate analyses revealed that HCC development was independently associated with age of ≥75 years (risk ratio [RR] = 35.16), α- fetoprotein levels of ≥6 ng/mL (RR = 40.30), and SWE results of ≥11 kPa (RR = 28.71).

**Conclusions:**

Our findings indicate that SWE may facilitate HCC surveillance after SVR and the identification of patients who have an increased risk of HCC after HCV clearance.

## Introduction

Hepatocellular carcinoma (HCC) is the fifth most common global cause of malignancy-related death [1], and a major cause of HCC is chronic hepatitis C virus (HCV) infection [2, 3]. Although achieving a sustained virological response (SVR) can help prevent HCC, the possibility of developing HCC remains approximately 1% per year after SVR [4–7]. In addition, a recent report revealed that the HCC recurrence rate is relatively high after SVR has been achieved using direct-acting antivirals agent [8].

Several recent reports have revealed that older age, male sex, advanced liver fibrosis, and high levels of α-fetoprotein (AFP) and alanine aminotransferase (ALT) are risk factors for developing HCC after achieving SVR [9–16].

Previous studies have also revealed that ultrasound elastography is a non-invasive tool for measuring liver stiffness (LS) and determining the stage of liver fibrosis [17–21]. Furthermore, ultrasound-based transient elastography is a simple and reliable tool for evaluating various liver diseases [22], although this approach does not provide two-dimensional images of the target structures and cannot be used for patients with ascites. Shear wave elastography (SWE) measures LS by quantifying the velocity of shear waves produced in the liver tissue and uses a normal B-mode ultrasound probe for measuring LS in real time. In addition, SWE imaging using the Aixplorer system provides a high frame-rate and may be able to provide more accurate scoring of fibrosis [23].

Several studies have revealed that LS decreases after patients have achieved SVR, compared to the pre-treatment value [24, 25]. Although the degree of fibrosis after SVR is a known risk factor for post-SVR HCC development, no studies have evaluated the association of fibrosis evaluated using shear wave elastography (SWE) with the development of post-SVR HCC. Therefore, this retrospective study evaluated the relationship between LS measured using SWE and HCC development after HCV eradication.

## Materials and methods

### Patients

This retrospective single-center study evaluated patients who achieved SVR after treatment using direct-acting antiviral agents (n = 107) or interferon-based treatment (n = 89) for HCV infection between February 2008 and May 2016. The exclusion criteria were patients with the hepatitis B virus surface antigen, antibodies against the human immunodeficiency virus, daily consumption of >40 g of ethanol, HCC before achieving SVR, or other liver diseases. HCC was ruled out by gadolinium-ethoxybenzyl-diethylenetriaminpentaacetic acid-enhanced magnetic resonance imaging or dynamic computed tomography before HCV treatment. Eligible patients were identified using their treatment records, and SVR was defined as being negative for HCV RNA at 24 weeks after the end of treatment (SVR24). The patients’ baseline clinical data were recorded at the start of therapy. Follow-up time was calculated from SVR24, and the follow-up was discontinued after the final visit for patients who did not develop HCC or the diagnosis of HCC for patients who developed HCC. The study’s protocol was approved by the ethics committee of Southern Tohoku Research Institute for Neuroscience (178) and conformed to the ethical guidelines of the 1975 Declaration of Helsinki. All patients provided informed consent before undergoing SWE. All data were fully anonymized before we accessed them.

### Liver stiffness measured using real-time SWE

SWE was performed before antiviral therapy and at SVR24. After conventional liver screening, real-time SWE was performed using the the Aixplorer ultrasound system with a convex broadband probe (SuperSonic Imagine S.A., Aix-en-Provence, France). The intercostal acoustic window was used to evaluate LS in the right liver lobe after a 6-h fast. The patient was placed in the supine position with their right arm raised and performed resting respiration for approximately 5s. The color box was placed at 1.5–3.0 cm below the liver capsule and in an area of parenchyma avoiding large vessels. In the color box, high elasticity is displayed in red and low elasticity is displayed in blue. A circular region of interest (ROI) with a diameter of 10 mm was defined in the color box to measure the mean, minimum, maximum, and standard deviation values for elasticity. The measurement was repeated three times, and the median value was used as the patient’s LS.

### Surveillance and HCC diagnosis

Patients were monitored after achieving SVR for 3–4 months and underwent ultrasonography plus laboratory testing, which evaluated complete blood cell count, aspartate aminotransferase (AST), ALT, AFP, and des-gamma-carboxy prothrombin (DCP). In addition, patients underwent magnetic resonance imaging once a year. In cases with a nodular lesion detected using ultrasonography or elevated levels of a tumor maker, the patient underwent magnetic resonance imaging and/or computed tomography. The imaging results were evaluated for the presence of HCC, which was radiologically diagnosed based on typical hemodynamic signs of classical HCC (considerable intensification during the arterial phase followed by a washout with corona-like peripheral enhancement in the equilibrium phase).

### Statistical analysis

Categorical variables were compared using the Kruskal-Wallis exact test or Fisher’s exact test. Median values of continuous variables were compared using the Mann-Whitney *U*-test. The rate of HCC development was evaluated using the Kaplan-Meier method and compared using the log-rank test. Univariate and multivariate analyses were performed to identify predictors of HCC using Cox proportional hazard regression models. All statistical tests were performed using IBM SPSS software (IBM Corp., Armonk NY), all tests were two-tailed, and differences with a P-value of <0.05 were considered statistically significant.

## Results

### Baseline characteristics and treatment effects

**Fig 1:**
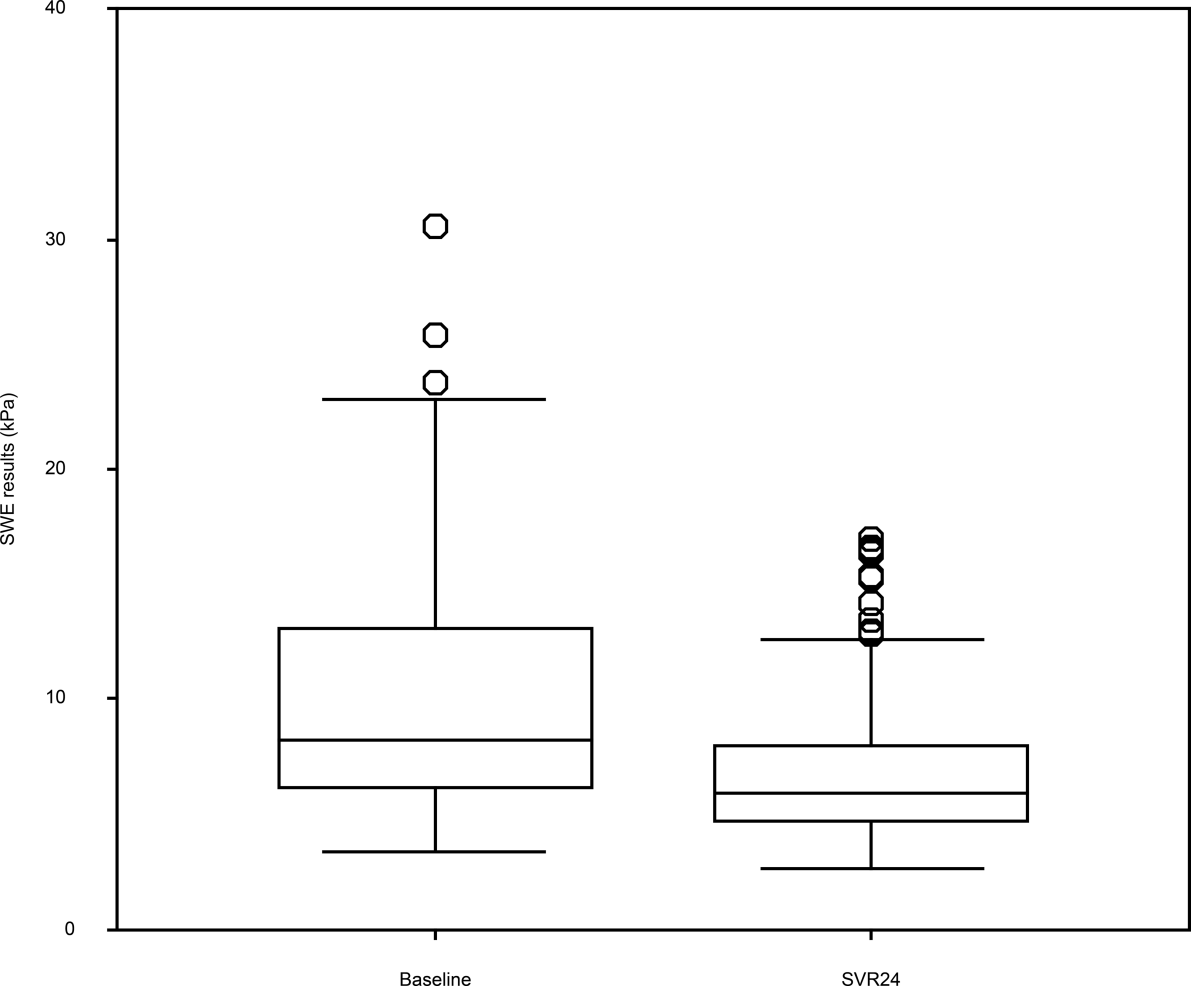
Shear wave elastography results at baseline and week 24 of the sustained virological response.

**Fig 2:**
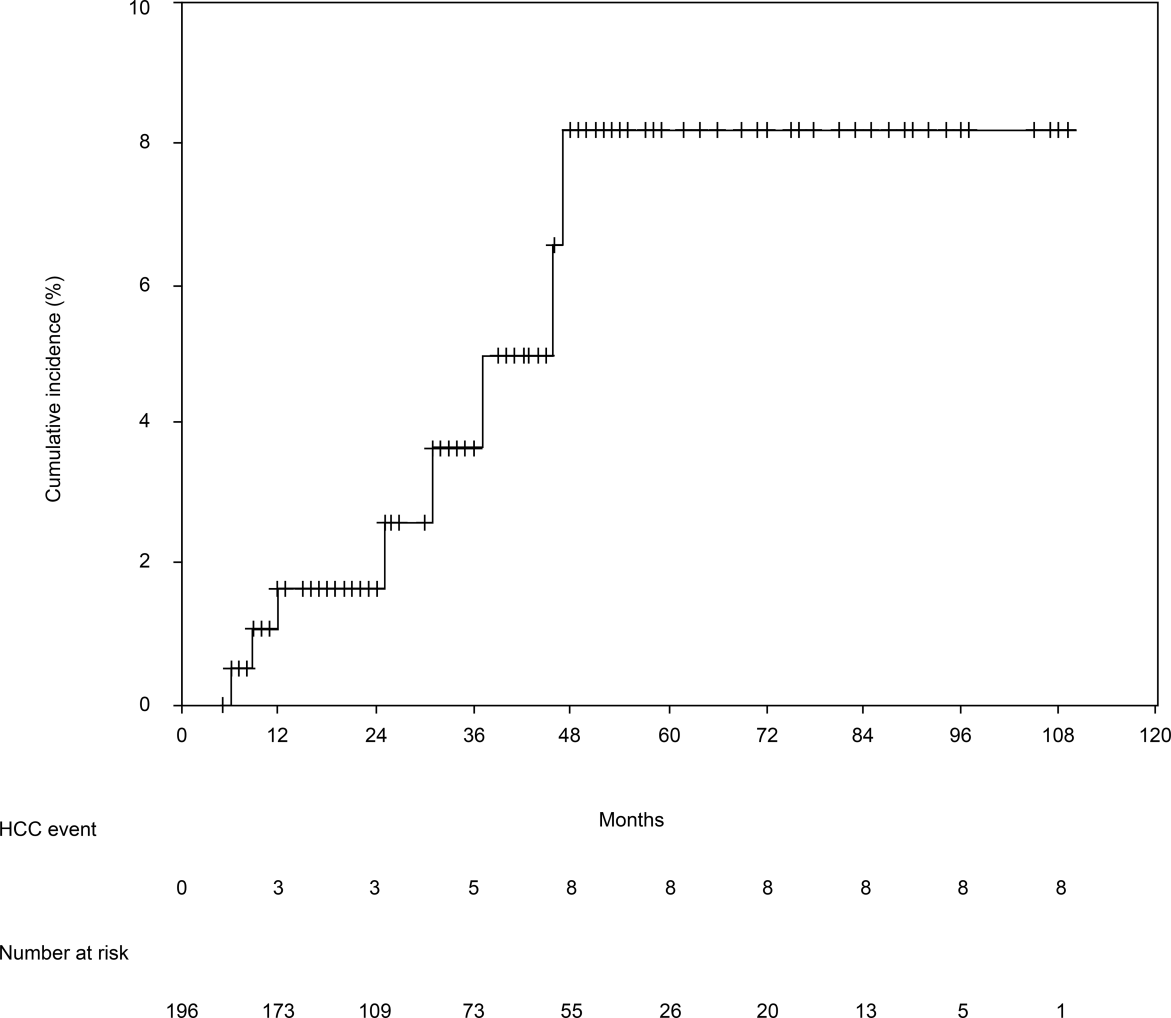
Cumulative incidence of hepatocellular carcinoma. The 12-month, 24-month, 36-month, and 48-month cumulative incidence rates for hepatocellular carcinoma were 1.6%, 1.6%, 3.6%, and 8.2%, respectively.

Table 1 shows the patients’ baseline characteristics before the antiviral therapy. The 196 patients included 89 men (45.4%) and 107 women (54.6%) with a median age of 62 years. The median follow-up period was 26 months (range: 5–109 months). Table 1 also shows the patients’ laboratory data, liver function, and SWE results at baseline and SVR24. Compared to baseline, the patients exhibited significant changes in the SVR24 values for ALT, AST, gamma-glutamyl transferase, albumin, platelet count, AFP, Fibrosis 4 index, and SWE. The shear wave elastography (SWE) results were 8.3 kPa (range: 3.4–36.2 kPa) at baseline and 5.9 kPa (range: 2.7–31.3 kPa) at week 24 of the sustained virological response (SVR24). This decrease was statistically significant (P < 0.001) (Fig 1). Among the 196 patients, 8 patients (4.1%) developed HCC after achieving SVR. The median time from SVR to HCC development was 28 months (range: 6–46 months). The cumulative incidences of HCC were 1.6% at 12 months, 1.6% at 24 months, 3.6% at 36 months, and 8.2% at 48 months (Fig 2).

**Table 1:** The patients’ baseline characteristics before antiviral therapy (n = 196).

	Baseline	SVR24	P-value
Age (years)^†^	62 (29–89)		
Sex (female/male)	107/89		
BMI (kg/m^2^)^†^	23 (14.8–35.5)		
Diabetes mellitus (yes/no)	22/174		
Genotype (1/2)	136/60		
HCV RNA (log10 IU/mL)^†^	6.2 (2.4–7.4)		
ALT (IU/L)^†^	39 (8–528)	16 (6–166)	<0.001
AST (IU/L)^†^	38 (12–269)	23 (10–136)	<0.001
GGTP (IU/L)^†^	36 (9–1,470)	21 (8–781)	<0.001
Albumin (g/dL)^†^	4.2 (2.1–4.9)	4.4 (2.6–5.2)	<0.001
Total bilirubin (mg/dL)^†^	0.63 (0.27–4.55)	0.68 (0.26–3.46)	0.127
Platelet count (×10^3^/μL)^†^	16.1 (5.3–49.1)	16.8 (5.7–46)	0.044
AFP (ng/mL)^†^	4.1 (8–123.7)	3.1 (0.6–16.9)	<0.001
DCP (mAU/mL)^†^	19 (10–207)	20 (10–72)	0.38
Fibrosis 4 index^†^	2.56 (0.39–12.13)	2.04 (0.42–10.88)	<0.001
SWE (kPa)^†^	8.3 (3.4–36.2)	5.9 (2.7–31.3)	<0.001
Follow-up duration (months)^†^	26 (5–109)		
Therapy (IFN-based/DAAs)	89/107		

^†^Values are expressed as median (range).

AFP, alpha fetoprotein; ALT, alanine aminotransferase; AST, aspartate aminotransferase; BMI, body mass index; DCP, des-gamma carboxyprothrombin; GGTP, gamma-glutamyl transpeptidase; HCV, hepatitis C virus; IFN, interferon; DAAs, direct-anting agents; SVR24, sustained virological response at week 24; SWE, shear wave elastography.

### Risk factors for HCC development after HCV eradication

**Fig 3:**
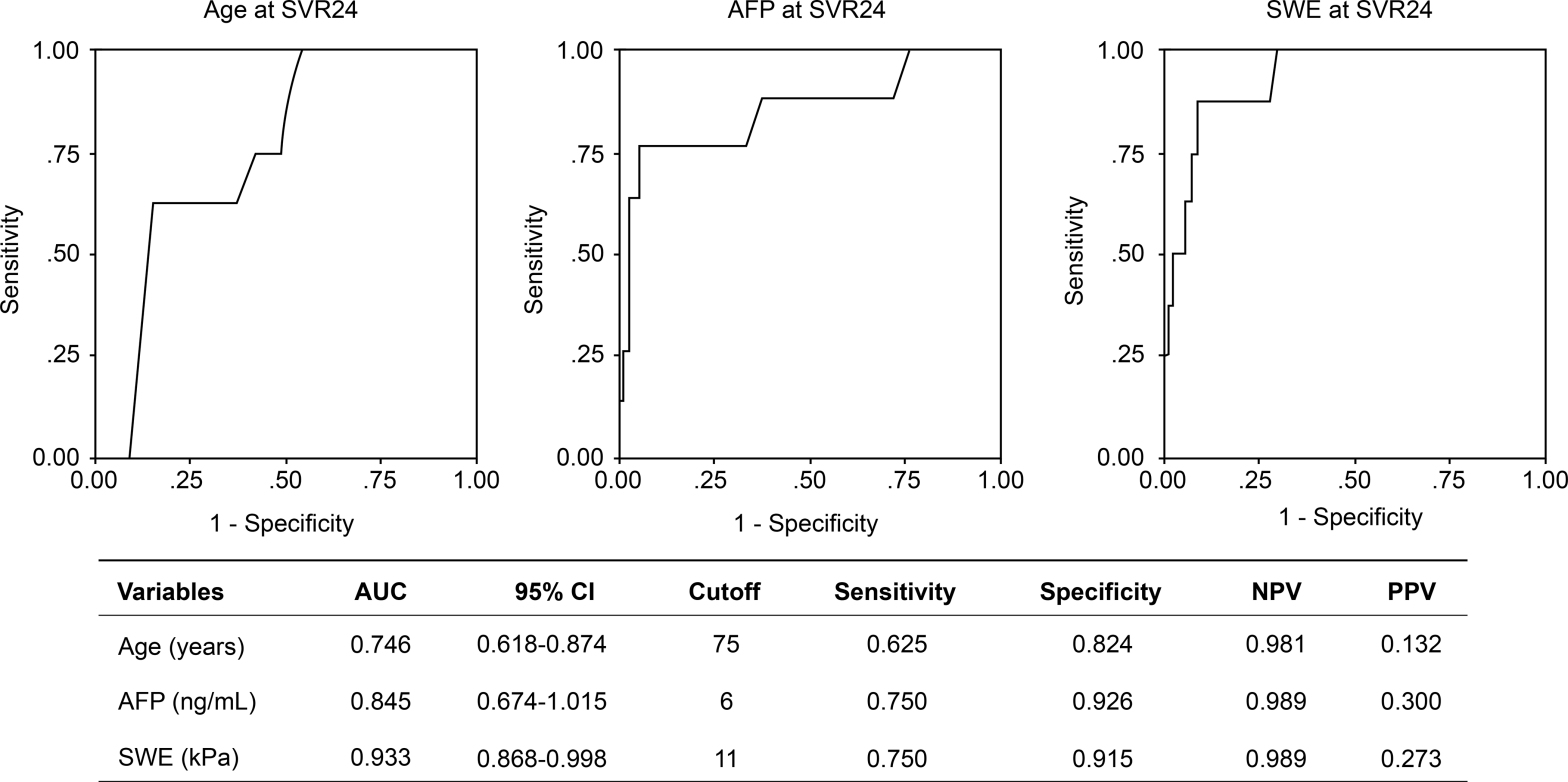
Predictive values for age, AFP, and SWE levels. ROC curve for predicting the development of HCC. Area under the ROC curve, 95% CI, cutoff value, sensitivity, specificity, negative predictive value (NPV), and positive predictive value are also shown.

The univariate analyses revealed that the development of HCC was associated with six variables at SVR24: age, BMI, albumin, platelet count, AFP, Fibrosis 4 index, and SWE. These factors were evaluated in the multivariate analyses, which revealed that HCC development was independently associated with age of ≥75 years (risk ratio [RR] = 35.16, P = 0.001), AFP levels of ≥6 ng/mL (RR = 43.30, P = 0.003), and SWE results of ≥11 kPa (RR = 28.71, P = 0.006). HCV genotype (1b or others) and therapy (Interferon-based or DAAs) were not associated with development of HCC (Table 2). The cutoff values for predicting the development of HCC were determined by a receiver operator characteristics (ROC) analysis (Fig 3). From the ROC analysis, ages ≥75 years, AFP levels of ≥6 ng/mL, and SWE results of ≥11 kPa were identified as cutoff values. Negative predictive values were high at 0.981 in age, 0.989 in AFP, and 0.989 in SWE. This result suggested patients with age, AFP, and SWE levels below these cutoff values were at a lower risk for the development of HCC.

**Table 2:** Univariate and multivariate analyses of risk factors for HCC development after SVR (n = 196).

Category	HR (95% CI)	P-value
Univariate analysis		
Age at SVR24 (years) <75 ≥75	1 7.83 (1.78–34.38)	0.006
Sex female male	1 1.45 (0.33–6.05)	0.648
BMI (kg/m^2^)^†^ <30 ≥30	1 7.50 (1.30–43.16)	0.024
AST (IU/L)^†^ <60 ≥60	1 8.81 (0.81–95.73)	0.074
ALT (IU/L)^†^ <60 ≥60	1 4.33 (0.46–41.01)	0.201
GGTP (IU/L)^†^ <40 ≥40	1 1.91 (0.37–9.92)	0.444
Albumin (g/dL)^†^ ≥3.9 <3.9	1 13.46 (3.02–60.09)	0.001
Total bilirubin (mg/dL)^†^ <0.8 ≥0.8	1 1.22 (0.28–5.27)	0.791
Platelet count (×10^3^/μL)^†^ ≥7 <7	1 31.00 (3.71–258.73)	0.002
AFP (ng/mL)^†^ <6 ≥6	1 37.28 (6.88–202.12)	<0.001
DCP (mAU/mL)^†^ <16 ≥16	1 1202.91 (0.00–3203)	0.819
Fibrosis 4 index^†^ <2.5 ≥2.5	1 14.21 (1.71–117.97)	0.014
SWE (kPa)^†^ <11 ≥11	1 32.24 (6.01–173.07)	<0.001
Diabetes No Yes	1 2.80 (0.53–14.82)	0.226
HCV genotype Others 1b	1 0.31 (0.37–2.53)	0.271
Therapy DAAs IFN-based	1 2.06 (0.48–8.88)	0.331
**Multivariate analysis**		
Age at SVR24 (years) <75 ≥75	1 35.16 (2.26–547.10)	0.011
AFP (ng/mL)^†^ <6 ≥6	1 43.30 (3.51–534.33)	0.003
SWE (kPa)^†^ <11 ≥11	1 28.71 (2.58–320.03)	0.006

^†^At week 24 of SVR.

AFP, alpha fetoprotein; ALT, alanine aminotransferase; AST, aspartate aminotransferase; BMI, body mass index; CI, confidence interval; DCP, des-gamma carboxyprothrombin; GGTP, gamma-glutamyl transpeptidase; HCC, hepatocellular carcinoma; HCV, hepatitis C virus; HR, hazard ratio; DAAs, direct-anting agents; IFN, interferon; SVR, sustained virological response; SWE, shear wave elastography.

### Cumulative incidences of HCC according to clinical factors

**Fig 4:**
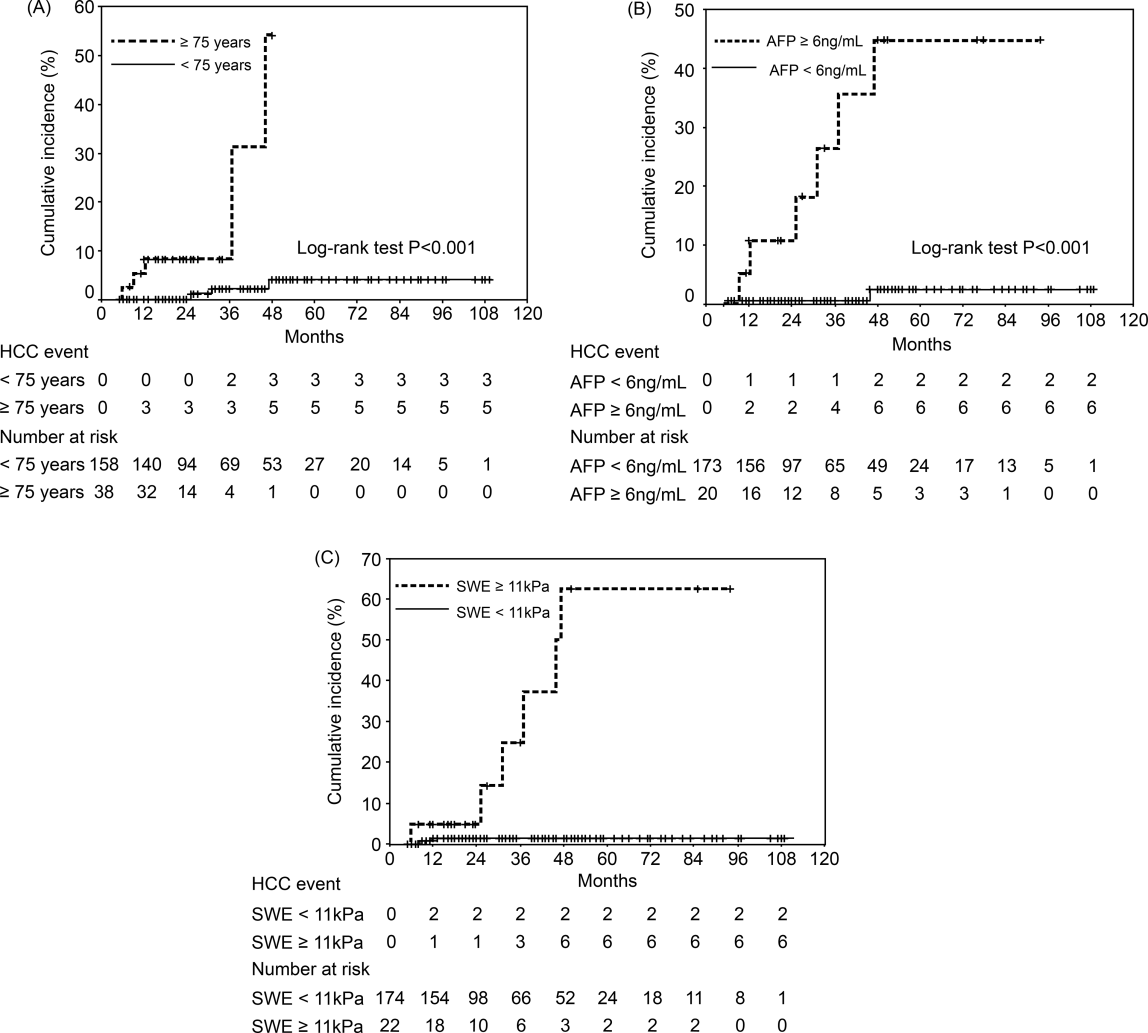
Incidences of hepatocellular carcinoma based on various factors. (a) Among patients with sustained virological response after 24 weeks (SVR24), the cumulative incidence of hepatocellular carcinoma (HCC) was significantly different between patients who were <75 years old and those ≥75 years old (P < 0.001). (b) Among patients with SVR24, the cumulative incidence of HCC was significantly different between patients with alpha fetoprotein (AFP) levels of <6 ng/mL and ≥6 ng/mL (P < 0.001). (c) Among patients with SVR24, the cumulative incidence of HCC was significantly different between patients with shear wave elastography (SWE) results of <11 kPa and ≥11 kPa at 24 weeks (P < 0.001).

Fig 4A shows the cumulative incidences of HCC according to age at SVR24. Among 158 patients who were <75 years old, the incidences were 0% at 12 months, 0% at 24 months, 2.2% at 36 months, and 4.0% at 48 months. Among 38 patients who were ≥75 years old, the incidences were 8.2% at 12 months, 8.2% at 24 months, 8.2% at 36 months, and 54.1% at 48 months (P < 0.001).

Fig 4B shows the cumulative incidences of HCC according to AFP levels at SVR24. Among 173 patients with AFP levels of <6 ng/mL, the incidences were 0.6% at 12 months, 0.6% at 24 months, 0.6% at 36 months, and 2.3% at 48 months. Among 20 patients with AFP levels of ≥6 ng/mL, the incidences were 10.8% at 12 months, 18.3% at 24 months, 18.3% at 36 months, and 44.8% at 48 months (P < 0.001).

Fig 4C shows the cumulative incidences of HCC according to SWE results at SVR24. Among 174 patients with SWE results of <11 kPa, the incidences were 1.3% at 12 months, 1.3% at 24 months, 1.3% at 36 months, and 1.3% at 48 months. Among 22 patients with SWE results of ≥11 kPa, the incidences were 4.6% at 12 months, 4.6% at 24 months, 24.8% at 36 months, and 62.4% at 48 months (P < 0.001).

## Discussion

This retrospective study evaluated risk factors or hepatocarcinogenesis after HCV eradication. The results indicate that the significant and independent risk factors for HCC development after SVR24 were age of ≥75 years, AFP levels of ≥6 ng/mL, and advanced liver fibrosis (SWE results of ≥11 kPa). Although the incidence of HCC in the present study was higher than the results of other studies, we believe this difference was related to the higher median age and progression of liver fibrosis in the present study. A recent study has also revealed that the incidences of HCC after SVR were similar between groups that received interferon-based therapy and interferon-free therapy (direct antiviral agents) [26], and this finding is consistent with our results.

Previous studies have revealed that the risk factors for HCC after SVR were older age, male sex, advanced liver fibrosis, and high levels of AFP and ALT [9–14]. In those studies, liver fibrosis was evaluated using liver biopsy, which is considered the gold standard for evaluating liver fibrosis. However, liver biopsy is also associated with severe complications, a risk of sampling error, and low patient acceptance. Real-time SWE may be a useful alternative, as a pilot study revealed that it provided an area under the receiver operating characteristic curve of 0.92 for differentiating absent and mild fibrosis (F0-F1) from significant fibrosis (≥F2) [23]. A recent retrospective study also revealed that patients with pretreatment LS measured using transient elastography (>12 kPa) had a high risk of developing HCC after SVR [27]. Those findings are consistent with our results.

In the present study, SWE results at SVR24 were significantly lower, compared to the baseline values. SWE findings are influenced by both tissue elasticity (fibrosis) and viscosity (inflammation). Because inflammation was improved at SVR24, it appears that SWE results at SVR24 provide a clear evaluation of liver fibrosis. Furthermore, a recent study suggested that SWE provides an accurate evaluation of liver fibrosis in patients who had achieved SVR [28].

Therefore, SWE with B-mode ultrasound may be useful for regularly measuring LS and screening for HCC after patients have achieved SVR. The present study has several limitations. First, the retrospective single-center design is associated with known risks of bias. Second, the follow-up period was not very long. Thus, prospective multi-center studies with longer follow-ups are needed to validate our findings. In addition, future studies should evaluate whether continued surveillance after SVR is a cost-effective approach.

In conclusion, HCC development after HCV eradication was independently associated with older age, elevated AFP levels, and high SWE values at SVR24. These findings indicate that SWE results may be an effective predictor of hepatocarcinogenesis after patients have achieved SVR, and may help reduce the reliance on liver biopsy to determine the degree of liver fibrosis and for risk of HCC development.

## Supporting information

S1 Table Data used in the study. (XLSX)
